# Supplementary material for: Functional ultrasound imaging of the brain reveals propagation of task-related brain activity in behaving primates
Source: Nat Commun. 2019 Mar 28;10:1400. doi: 10.1038/s41467-019-09349-w (PMC6438968; doi:10.1038/s41467-019-09349-w)
Supplement: Supplementary file 2 — Supplementary Information [file 41467_2019_9349_MOESM2_ESM.pdf]

Functional ultrasound imaging of the brain reveals propagation of task-related brain activity in behaving primates.

Dizeux et al. 2019

Supplementary figures

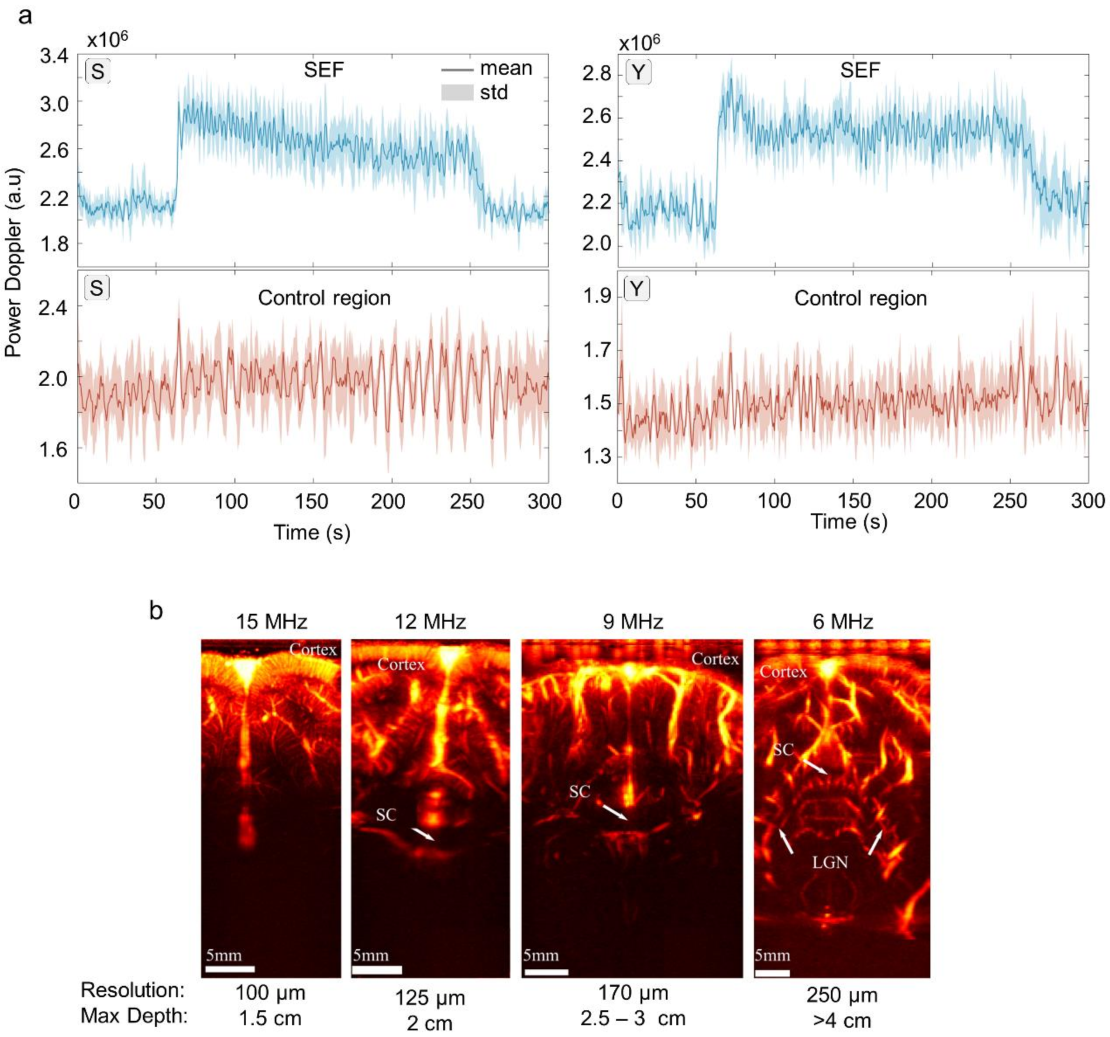

Supplementary Figure 1: Acquisition reproducibility and Probe frequency. **a** Average and standard deviation of five consecutive acquisitions for both animals in the SEF and control region for large ROIs (>200 pixels). **b** fUS imaging of the same visual cortex of a non-human primate seen with a different ultrasonic probe used at their central frequency. The probe used for the present study was the one at 15 MHz. LGN = lateral geniculate nucleus, SC = superior colliculus.

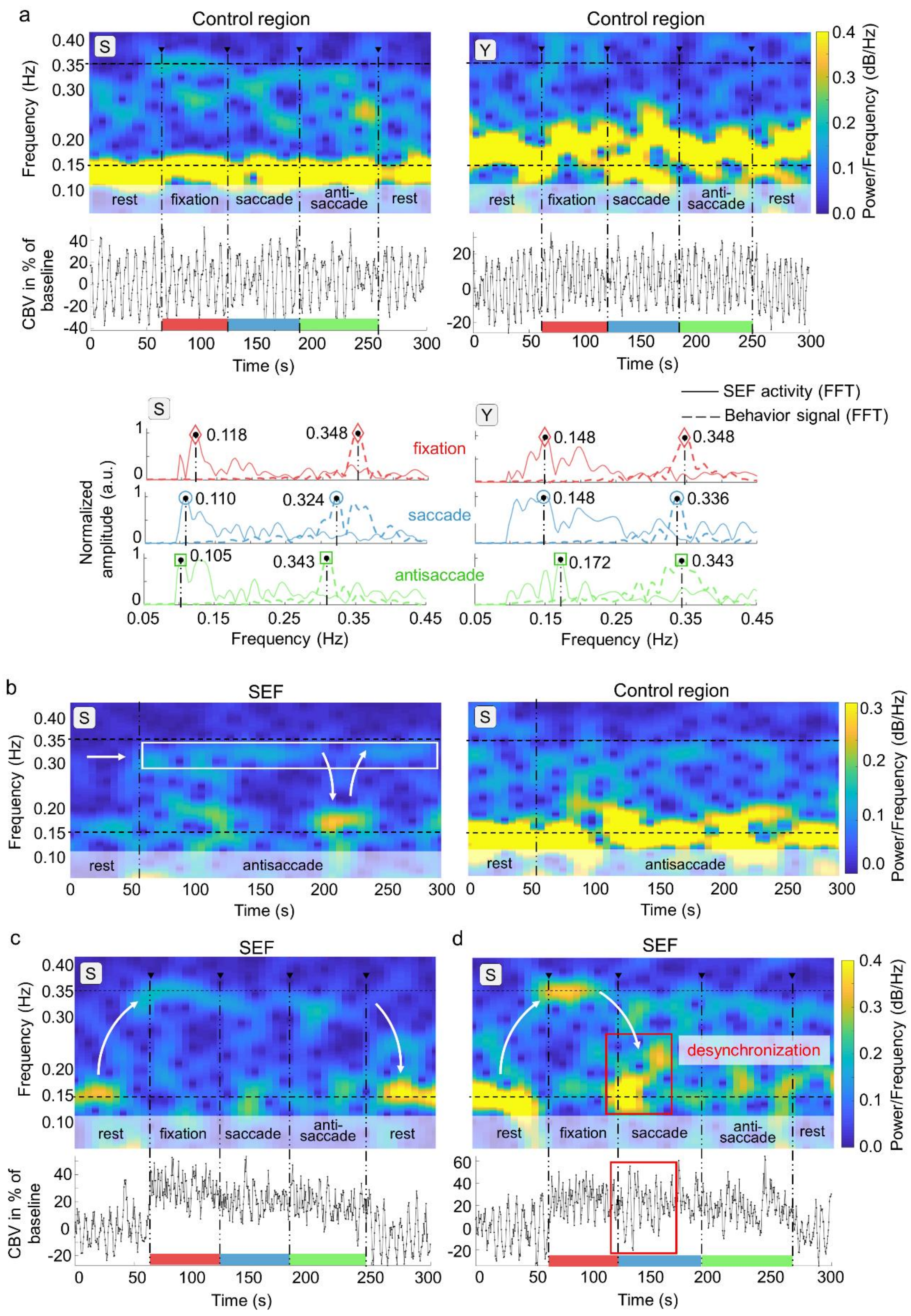

Supplementary Figure 2: Different examples of CBV spectrogram in specific cortical region and condition. **a** For both animals, CBV oscillations in the control region, in a range between 0.1 and 0.2 Hz, were not related to behavior activity (~0.35 Hz). **b** Spectrograms in SEF and control region during experiment with only one type of visual task (antisaccade). **c** In some acquisitions, when entering in the working phase, CBV oscillations in the SEF shifted from 0.15 Hz to 0.35 Hz and came back to the initial state at 0.15 Hz after stopping visual tasks. **d** An example of the desynchronization of SEF activity during the visual task.

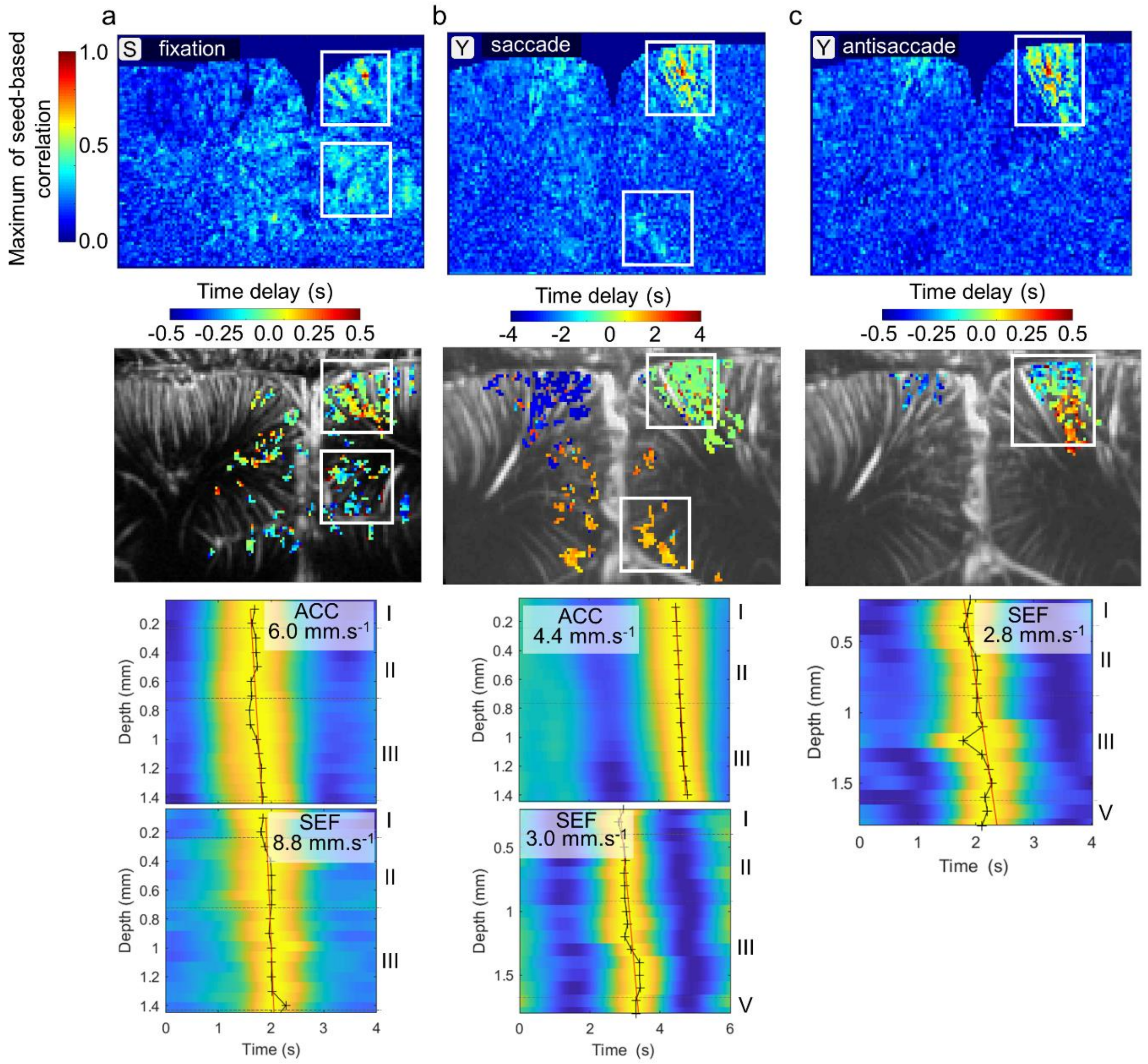

Supplementary Figure 3: Propagation of SEF-correlated information. Maximum of seed-based correlation maps with related time delays between cortical regions of interest (ACC, SEF) and the propagation speed of SEF-correlated information for 3 different cases. **a** Configuration found for nearly every acquisition for animal S. SEF-correlated information goes through the ACC before reaching the SEF. **b** An example for animal Y, for which the SEF-correlated information passes through the SEF before reaching the ACC. **c** In some cases, no SEF-correlated information was detected in the ACC. Cortical layers were delineated based on reference[55].

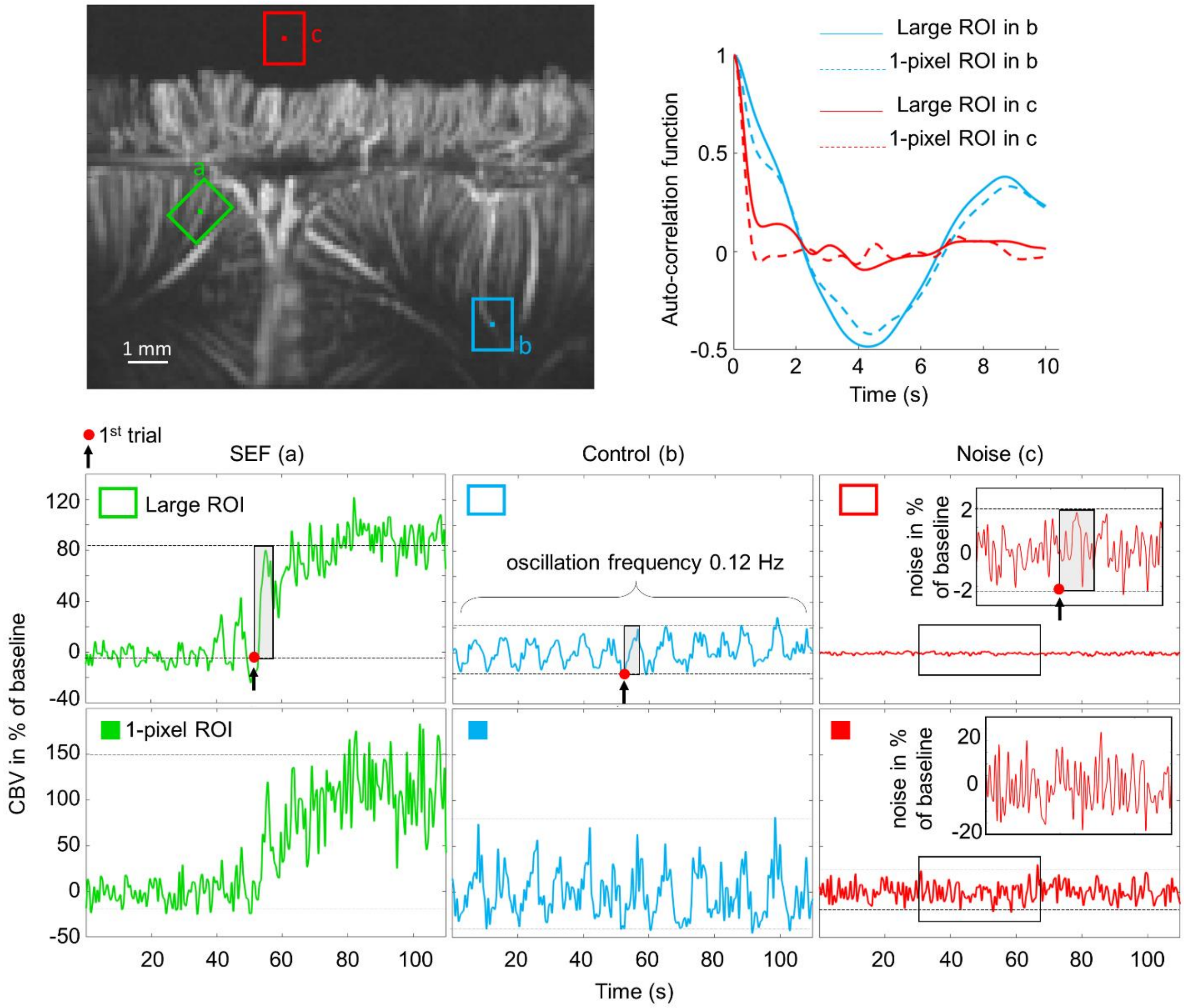

Supplementary Figure 4: The high sensitivity of fUS imaging. In a single Doppler movie it was possible to either extract CBV fluctuations in SEF (ROI a) related to visual activity, spontaneous coherent oscillations in control region (ROI b) and noise signal outside of the brain (ROI c). The auto-correlation function revealed a typical white gaussian noise concerning signal outside of the brain and clear coherent 0.12Hz CBV oscillations within control region.
